# Mean nocturnal baseline impedance and endoscopic mucosal impedance measurements in patients with eosinophilic esophagitis: a new tool for follow up and management?

**DOI:** 10.1007/s13304-022-01331-4

**Published:** 2022-07-15

**Authors:** Sergeev Ilia, Velosa Monica, Sifrim Daniel

**Affiliations:** Barts and the London School of Medicine and Dentistry, Wingate Institute of Neurogastroenterology, Queen Mary University of London, London, UK

**Keywords:** Eosinophilic esophagitis, Mean nocturnal baseline impedance (MNBI), Mucosal impedance

## Abstract

Eosinophilic esophagitis (EoE) is the second most common cause of chronic esophageal inflammation after GERD, with increasing incidence and prevalence across all age groups. Since current diagnosis and follow up of EoE require endoscopy with biopsies, there is an increased interest in non or less invasive tests for its diagnosis and follow up. Baseline mucosal impedance measurement allows evaluation of mucosal barrier properties and is widely accepted as an adjunct method in GERD diagnosis. As EoE is associated with increased mucosal permeability, the use of baseline impedance to evaluate mucosal integrity has been investigated in several studies. It was found that baseline mucosal impedance, measured either during 24 h reflux monitoring or during endoscopy, was significantly lower in all parts of the esophagus in EoE patients. Impedance measurement correlated with eosinophil counts on biopsies, offering a tool to monitor treatment response. Additionally, baseline impedance patterns differed between those responding to proton pump inhibitor (PPI) treatment and those resistant to PPI, potentially allowing to tailor future treatment to the individual patient. In summary, baseline impedance measurement offers a potential tool for diagnosis and follow up in EoE. Its exact place in EoE treatment is yet to be determined and requires further future studies.

Eosinophilic esophagitis (EoE) is a chronic, immune-mediated or antigen-mediated esophageal disease, second only to GERD as a cause of chronic esophageal inflammation. Incidence and prevalence of EoE is increasing in both children and adults [1]. The exact cause of EoE is currently unknown, but a strong association with atopic diseases such as asthma and atopic dermatitis is well established [2]. In fact, a similar inflammatory pattern involving a local T-helper 2 type inflammation is present in the esophagus of patients with EoE, patients with asthma and those with atopic dermatitis [3]. The diagnosis is based on the presence of increased number of eosinophils in esophageal mucosal biopsies. Patients with EoE can be either asymptomatic, or present with dysphagia, food impaction or refractory GERD, and can progress to esophageal fibrosis and stricture formation if left untreated [4]. Thus, surveillance is paramount to prevent long term complications. As both the diagnosis and the treatment response assessment in EoE require an endoscopic procedure with biopsies, there is an increased interest in non or less invasive for its diagnosis and follow up.

Intraluminal impedance monitoring measures changes in conductivity to an alternating electrical current along electrodes mounted on a catheter, which in a tubular organ such as the esophagus, in the absence of swallowing or reflux, are in close contact with the mucosa. The electrical impedance, expressed in ohms, is the equivalent to the resistance to the electrical current [5]. Multichannel intraluminal impedance (MII) can thus detect bolus movement by measuring the direction of changes in electrical resistance, caused by fluid or gas: liquid bolus with more ions will have lower impedance than the baseline, while gas with fewer ions will have a higher value [6]. Between bolus movement the impedance levels return to a baseline level, which is dependent on mucosal characteristics of the collapsed esophageal wall [7](Fig. 1).

**Fig. 1:**
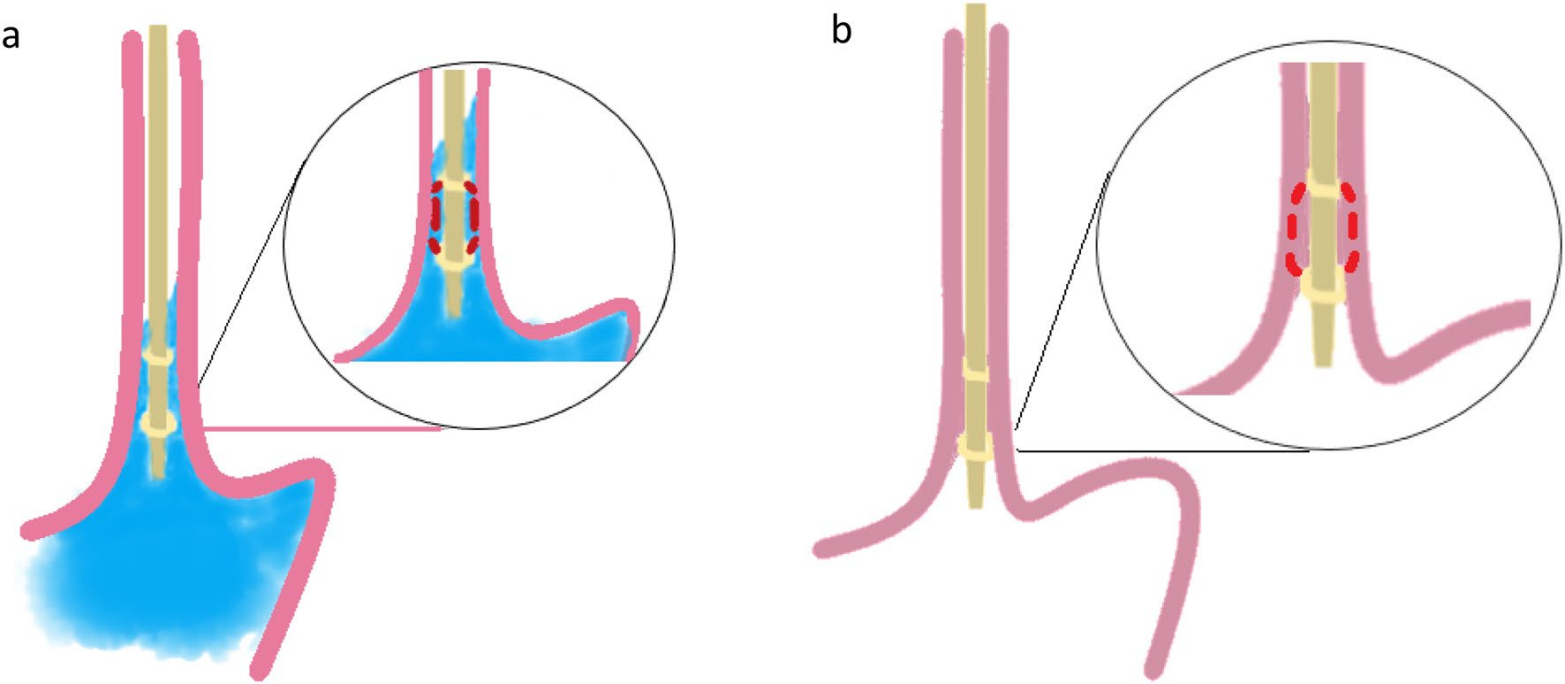
Esophageal impedance measurement with liquid in esophagus (**a**) and with collapsed esophageal wall (**b**)

Low baseline impedance measurements have been used as a marker for impaired mucosal integrity, a finding initially demonstrated in acid-induced heartburn in healthy volunteers and in GERD patients [7]. Moreover, lower baseline esophageal impedance in GERD patients was found to correlate with higher acid exposure time (AET), and increase in impedance following proton pump inhibitor (PPI) treatment was noted in those patients [8]. Woodland et al [9] found that mean baseline mucosal impedance levels could help differentiate between non-erosive reflux disease (NERD) and those with functional heartburn. Similar results were found in a study by Kandulski et al [10], demonstrating that baseline impedance in lower esophagus can differentiate between functional heartburn, GERD and NERD. Likewise, patients with Barrett’s mucosa were found to have significantly lower baseline impedance compared to patients with normal mucosa [11].Initially, baseline impedance was calculated manually for a 6-h period, excluding reflux episodes and swallow events. The concept of Mean Nocturnal Baseline Impedance (MNBI) was introduced by Martinucci et al. [12]. While still requiring manual calculations, MNBI measurement consisted of evaluating 10-min periods at 3 different timepoints during night, and gave results similar to traditional baseline impedance measurements. Frazzoni et al. demonstrated that the use of MNBI and additional measure of post-swallow peristaltic wave index (PSPW) increased the diagnostic yield of impedance pH monitoring of patients with reflux disease, especially NERD [13]. In another study, the same group demonstrated the ability of MNBI and PSPW index to distinguish between reflux and functional heartburn patients in PPI-refractory patients [14]. Further studies demonstrated that MNBI correlated with reflux burden [13, 15] and both symptomatic relief [15, 16]and endoscopic healing [16] following therapy. The normal values for mucosal impedance were found to have significant regional variability, possibly influenced by various factors such as genetics, BMI and meal composition [17]. Recently, a simplified method for MNBI measurement was introduced, allowing fast and accurate mucosal impedance assessment [18](Fig. 2). In the Lyon consensus published in 2018 [19], the use of MNBI was endorsed as an adjunct for GERD diagnosis in borderline cases.

**Fig. 2:**
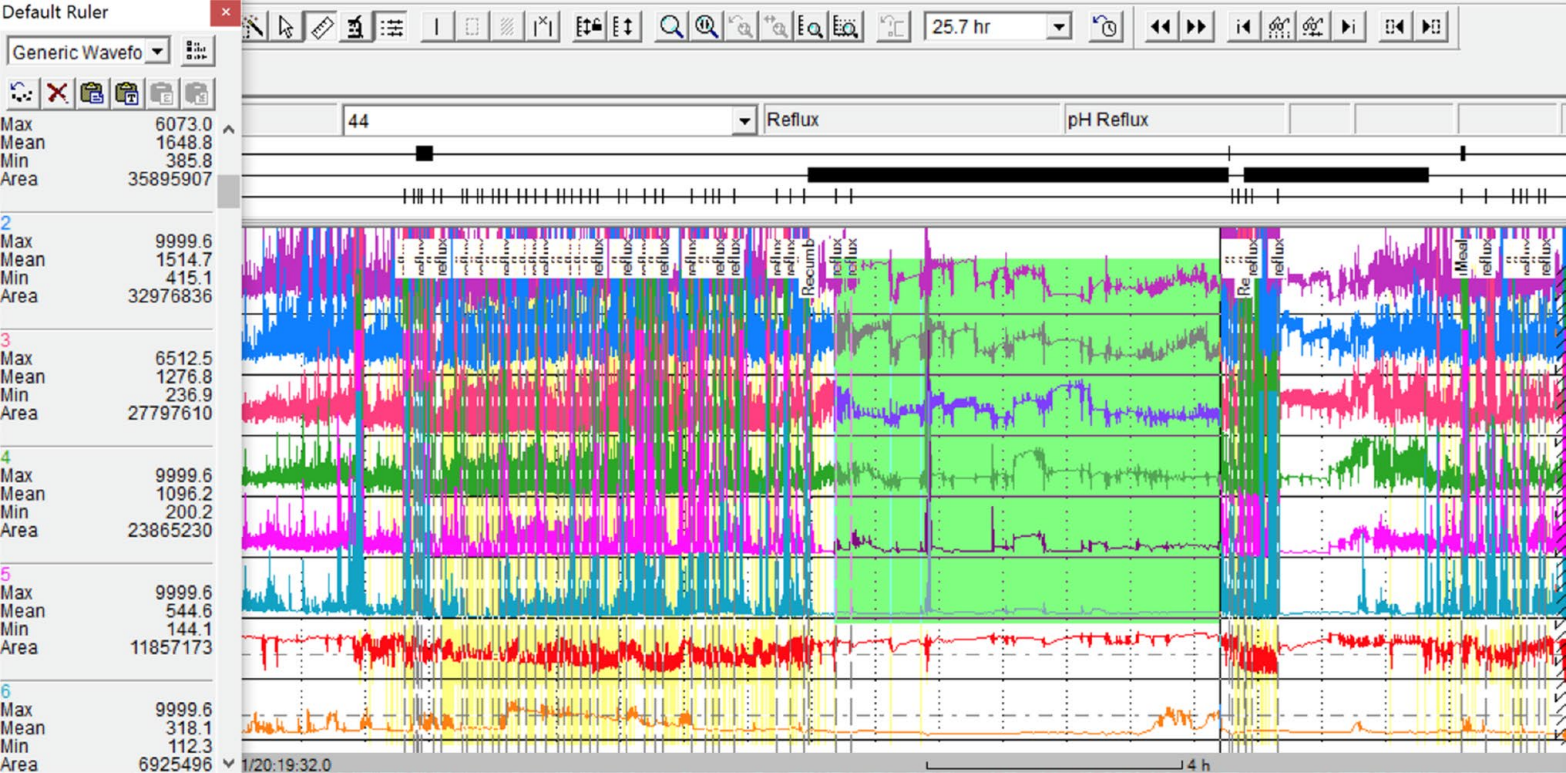
Simplified measurement of baseline impedance (shown patient with low baseline impedance in lower channels, typical for GERD)

Several previous studies demonstrated specific mucosal alterations in EoE that result in increased mucosal permeability. The dilation of the intercellular space in EoE is well documented and is considered one of the histologic hallmarks for the disease [20]. Previous studies demonstrated reduced expression of intercellular junction proteins [21, 22] and adhesion molecules [23], with inverse correlation between junction protein expression and dilation of the intercellular space [24]. In addition, higher expression of proteases with lower expression of protease inhibitors was demonstrated in EoE mucosa [22], potentially weakening the tight junctions between cells and increasing permeability. Using Ussing chambers in vitro, Warnes et al [25] demonstrated lower transepithelial resistance and increased mucosal permeability in EoE patients.

In their study in 2013, Van Rhijn et al [26] investigated baseline impedance in EoE patients and its relationship with acid exposure. Ambulatory 24 h pH-impedance was performed in EoE and healthy controls with matched acid exposure, and impedance levels in proximal, middle and distal esophagus were compared between cohorts. Reflux characteristics including proximal reflux extent were similar between cohorts as per study design. Baseline impedance levels in EoE patients were markedly lower compared to healthy controls in the distal, middle and proximal esophagus. Baseline impedance decreased from proximal to distal in healthy subjects. However, EoE patients had decreased baseline impedance throughout the esophagus, without such gradient (Fig. 3).

**Fig. 3:**
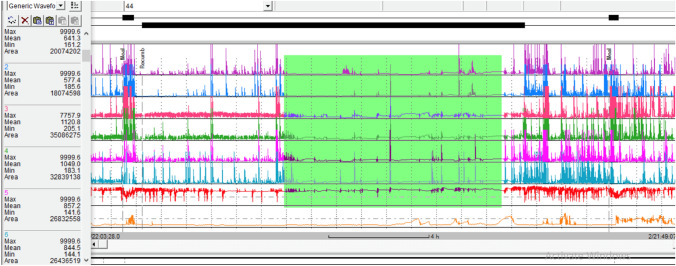
Low baseline impedance in all channels, typical in EoE patients

As high prevalence of EoE in patients with esophageal atresia was reported, Pesce et al. [27] investigated the use of MNBI in pediatric patients with esophageal atresia. The diagnosis of EoE in those patients is often challenging due to symptom overlap with GERD and presence of dysphagia due to esophageal dysmotility secondary to atresia and its repair. The delay in diagnosis might put the children in risk for EoE complications. It was found that patients with both esophageal atresia and EoE had lower impedance values compared with GERD patients or EoE patients without history of atresia repair. The authors suggested that MII-pH could be valuable in selecting which patients with esophageal atresia should undergo endoscopic examination.

In addition to the measurement of MNBI during MII-pH monitoring, other methods for impedance measurement have been investigated in EoE. The question whether direct mucosal impedance could differentiate between GERD and non-GERD patients has been explored by Ates et al [28], using a method of direct mucosal impedance measurement using a specific probe during endoscopy. The authors were able to demonstrate characteristic impedance patterns for GERD, non-GERD and EoE patients, allowing discrimination between these conditions. In another study, Katzka et al [29] demonstrated a correlation between mucosal impedance and eosinophil count on biopsies, potentially allowing to differentiate between active and non-active EoE patients. A similar ability to distinguish between active and in-remission EoE patents based on mucosal impedance was demonstrated by Warners et al [25]. In their study, both mucosal integrity measured by electrical tissue spectroscopy (ETIS) in vivo, and by transepithelial electrical resistance and transepithelial molecule-flux in vitro, correlated with esophageal eosinophilia. Mucosal impedance was similar between patients treated with diet and those treated with fluticasone.

Investigating the influence of reflux on EoE, Frazzoni et al. [30] demonstrated that off-PPI MNBI was able to distinguish between PPI responsive to PPI-refractory EoE. Those with PPI refractory EoE had significantly lower MNBI gradients between mid and lower esophagus and had lower MNBI improvement following PPI treatment. In a follow up study, greater mucosal damage, as measured by lower MNBI, was found in lower esophagus of PPI responsive patients, when compared to PPI-refractory ones [31].

In conclusion, measurement of mucosal baseline impedance during 24 h reflux monitoring or instant mucosal impedance during endoscopy offers a potential tool for diagnosis and follow up in EoE. Its exact place in EoE treatment is yet to be determined and requires further future studies.

## Declarations

### Research involving human and animal partcipants

As a review article, no have human or animal participants were involved, and therefore no ethical committee approval nor informed consent were sought.
